# Influence of risedronate on orthodontic tooth movement in rodents: a systematic review and case report

**DOI:** 10.1590/2177-6709.28.6.e2322280.oar

**Published:** 2024-01-08

**Authors:** Roberta Magalhães MIRANDA, Juliana Lourdes FERNANDES, Mariana de Souza SANTOS, Humberto JÁCOME-SANTOS, Roselaine Moreira Coelho MILAGRES, Henrique PRETTI, Lucas Guimarães ABREU, Soraia MACARI

**Affiliations:** 1 Universidade Federal de Minas Gerais, Faculdade de Odontologia, Departamento de Odontologia Restauradora (Belo Horizonte/MG, Brazil).; 2 Universidade Federal de Minas Gerais, Instituto de Ciências Biológicas, Departamento de Fisiologia e Biofísica (Belo Horizonte/MG, Brazil).; 3 Universidade Federal de Minas Gerais, Faculdade de Odontologia, Departamento de Patologia e Cirurgia Odontológica (Belo Horizonte/MG, Brazil).; 4 Universidade Federal de Minas Gerais, Faculdade de Odontologia, Departamento de Saúde Bucal da Criança e do Adolescente (Belo Horizonte/MG, Brazil).

**Keywords:** Risedronic acid, Tooth movement techniques, Osteoporosis

## Abstract

**Introduction:**

Bisphosphonates have an inhibitory impact on osteoclastic activity, reducing bone resorption. However, the influence of risedronate on tooth movement is not well-defined.

**Objective:**

This systematic review assessed the effect of risedronate intake on orthodontic tooth movement. A case report was also provided.

**Methods:**

Two independent reviewers searched six databases (PubMed, Web of Science, Ovid, Lilacs, Scopus, and Open Grey). The searches were carried out in April/2020, and an update was set in place in June/2023. Therefore, the searches considered a timeline from the databases’ inception date until June/2023, with no publication date and/or language restrictions. The clinical question focused on evaluating the orthodontic tooth movement and relapse movement (Outcome) in animals (Population) exposed to risedronate (Exposure), compared to control groups (Comparison). The Preferred Reporting Items for Systematic Review and Meta-Analysis (PRISMA) guidelines were applied, and the protocol was registered in PROSPERO (CRD42020168581). The risk of bias was determined using the Systematic Review Centre for Laboratory Animal Experimentation protocol (SYRCLE).

**Results:**

Two studies in rats and one in guinea pigs were included in the systematic review. The studies reported a decrease in orthodontic tooth movement, a reduction in the relapse movement, and a reduced number of positive tartrate-resistant acid phosphatase (TRAP) cells, with a significantly reduced number of bone gaps after the administration of risedronate in rats. A case report illustrated the effects of risedronate administration in one patient.

**Conclusion:**

Based on the systematic review, risedronate seems to impair orthodontic tooth movement and relapse due to a decrease in bone resorption cells.

## RESUMO

**Introdução:**

Os bifosfonatos têm um impacto inibitório na atividade osteoclástica, reduzindo a reabsorção óssea. No entanto, a influência do risedronato no movimento dentário não está bem definida.

**Objetivo:**

Esta revisão sistemática avaliou o efeito do uso de risedronato no movimento ortodôntico dos dentes. Um relato de caso também é apresentado.

**Métodos:**

Dois revisores independentes pesquisaram seis bases de dados (PubMed, Web of Science, Ovid, Lilacs, Scopus e Open Grey), considerando o período de abril de 2020 até junho de 2023, sem restrições de data e/ou idioma de publicação. A questão clínica focou em avaliar o movimento ortodôntico dos dentes e movimento de recidiva (resultado) em animais (população) expostos ao risedronato (exposição) em comparação com grupos de controle (comparação). Foram aplicadas as Diretrizes Preferenciais para Revisão Sistemática e Metanálise (PRISMA) e um protocolo foi registrado no PROSPERO (CRD42020168581). O risco de viés foi determinado utilizando o protocolo do Centro de Revisão Sistemática para Experimentação em Animais de Laboratório (SYRCLE).

**Resultados:**

Dois estudos em ratos e um em porquinhos-da-índia foram incluídos na revisão sistemática. Os estudos relataram uma diminuição no movimento ortodôntico dos dentes, uma redução no movimento de recidiva e um número reduzido de células positivas à fosfatase ácida tartarato-resistente (TRAP) com um número significativamente reduzido de falhas ósseas após a administração de risedronato em ratos. Um relato de caso ilustrou os efeitos da administração de risedronato em uma paciente.

**Conclusão:**

Com base na revisão sistemática, o risedronato parece interferir no movimento ortodôntico dos dentes e na recidiva devido a uma diminuição nas células de reabsorção óssea.

## INTRODUCTION

Orthodontic treatment can enhance the quality of life of individuals and improve gnathic function, providing better occlusion and esthetics.^1^ Tooth movement occurs through bone remodeling,^2^ which is only possible because of the sequential activity of osteoclasts (bone resorption) in areas of pressure, and osteoblasts (bone formation) in areas of tension.^2^ Factors that affect tooth movement during orthodontic treatment have been widely studied, and the use of drugs that alter bone turnover, such as bisphosphonates, has been investigated.^3^

Bisphosphonates are anti-resorptive drugs used to treat or prevent bone disorders, such as osteoporosis.^4^ Their mechanism of action includes an inhibitory effect on osteoclastic activity, which consequently reduces bone resorption.^5^ ^,^ ^6^ Bisphosphonates are used for the treatment of several osseous disorders.^4^ ^,^ ^7^ The main subtypes of bisphosphonates are alendronate, ibandronate, risedronate, pamidronate, clodronate, and zoledronic acid. The risedronate acid, or risedronate, is a pyridinyl bisphosphonate with a specific indication for the treatment of osteoporosis and to prevent fractures in postmenopausal women.^8^ ^,^ ^9^

Prospective studies have demonstrated that risedronate reduces the risk of vertebral, non-vertebral, and hip fractures.^5^ ^,^ ^6^ ^,^ ^9^ Although the drug may be associated with bisphosphonate-related osteonecrosis of the jaw in women,^10^ risedronate has a marked effect in reducing the prevalence of periapical lesions,^11^ as well as in increasing bone density in rats with glucocorticoid-induced osteoporosis.^12^ The effects of risedronate on mechanically-induced tooth movement, however, have not been well-defined, due to the absence of studies in human subjects and methodological discrepancies in experimental models. The present study aimed to compile, in a systematic review, data published specifically about risedronate, to verify its effects on orthodontic tooth movement. A case report of a postmenopausal patient who took risedronate before and while performing the orthodontic treatment is also provided. The present data associated with the clinical case may alert orthodontists about the need for rigorous anamnesis and clinical examination before orthodontic treatment, and carefully consider all medications used by their patients that may alter bone remodeling and, consequently, orthodontic treatment.

## MATERIAL AND METHODS

### ELIGIBILITY CRITERIA

The question proposed was *“Does the systemic or local administration of risedronate affect orthodontic tooth movement in animals?”*. Eligibility criteria included original studies that assessed orthodontic tooth movement and relapse movement in animals who were undergoing treatment with risedronate (administered orally or injected, with a systemic or local effect), and the administration of risedronate after treatment, in order to assess factors related to stability. A control group was required as inclusion criteria. All treatment schedules, frequencies, and dosages were eligible for inclusion. Case studies, case series, comments, letters to the editor, narrative reviews, and studies that evaluated histological or cytological aspects, but did not evaluate tooth movement, were excluded. The following PECO question was applied:

» P (Participants) = animals.
» E (Exposure) = systematic or local administration of risedronate.
» C (Comparison) = no systematic or local administration of risedronate.
» O (Outcome) = tooth movement.

### SOURCES OF INFORMATION AND SEARCH STRATEGIES

The following electronic databases were used: PubMed, Web of Science, Ovid, Lilacs, and Scopus. A search in Google Scholar and a search of the gray literature in Open Gray were also conducted. No publication dates or language restrictions were applied. The searches were carried out in April/2020, and an update was set in place in June/2023. Therefore, the searches considered a timeline from the databases’ inception date until June/2023. The complete search strategy was based on the following search terms linked with Boolean operators: Atelvia OR “Risedronate Sodium” OR “Risedronic Acid Monosodium Salt” OR Actonel OR Risedronate OR “Bisphosphonate Risedronate Sodium” OR Bisphosphonate OR 2-(3-pyridinyl)-1-hydroxyethylidene-bisphosphonate OR 2-(3-pyridinyl)-1-hydroxyethylidenebisphosphonate AND “Tooth Movement Technique” OR “Tooth Movement Techniques” OR “Orthodontic Tooth Movement” OR “Orthodontic Tooth Movements” OR “Tooth Up righting” OR “Minor Tooth Movement” OR “Minor Tooth Movements” OR “Tooth Intrusion” OR “Tooth Intrusions” OR “Tooth Depression” OR “Tooth Depressions” OR “Orthodontic Treatment” OR “Orthodontic Therapy” OR “Orthodontic Movement” OR “Tooth Movement”. The search strategies for the other databases are shown in Appendix 1.

### SELECTION OF STUDIES

Two authors independently reviewed the references retrieved in the searches, examining the titles and/or abstracts of the studies. When the abstracts of the articles were unavailable or did not provide sufficient information to reach a decision on inclusion or exclusion, the full text was then assessed. References that met the eligibility criteria were included. Any disagreements between authors about the eligibility of specific studies were resolved through a discussion with a third reviewer.

### DATA EXTRACTION AND ITEMS EXTRACTED

Data were extracted and incorporated into three tables in Excel^®^. The following data were extracted: authors (year, country), study design, study subjects, mean age, study groups, study duration, and primary assessment methods. The data also included all of the characteristics of the experiments, such as duration, applied force, other displacements, and statistical analysis, and information on the results and conclusions of the included studies. Data extraction was performed by two authors of the systematic review, independently, and the discrepancies were identified and resolved through discussion. 

### RISK OF BIAS ASSESSMENT

Two independent authors used the SYRCLE risk of bias tool^13^ to assess the risk of bias. Any disagreement between the two authors was resolved with a third party.

### EFFECT MEASURE

Information on any effect measure concerning the influence of risedronate on orthodontic tooth movement in rodents was collected.

### SYNTHESIS OF RESULTS

Data of the included studies were heterogeneous, and any attempt to perform a quantitative analysis was unfeasible in the period of April/2020 to June/2023.

## RESULTS

### STUDY SELECTION

**Figure 1:**
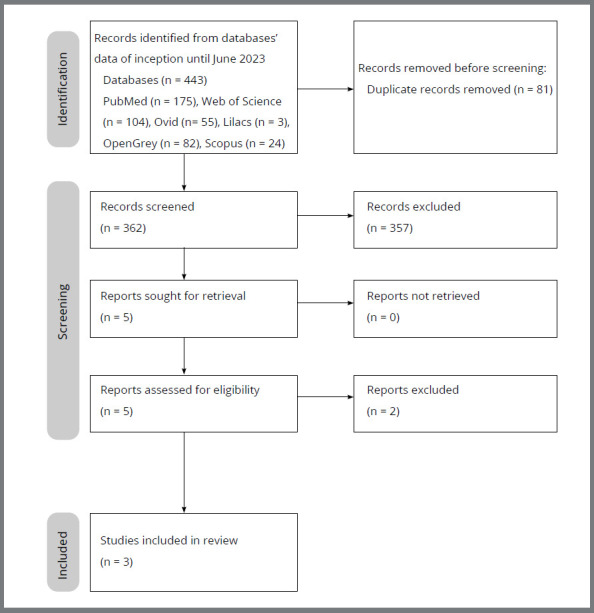
Flow diagram of study selection.

The database searches retrieved 443 references. After the removal of 81 duplicate records, 362 titles and/or abstracts were examined. Three hundred fifty-seven studies were excluded due to not meeting the eligibility criteria. Five studies were selected for full-text evaluation. Two studies were excluded because only histological or cytological aspects had been evaluated. Thus, three studies with animals were included in this systematic review (Adachi et al.^14^, 1994; Wu et al.^15^, 2019; Utari et al.^16^, 2021). A flow diagram of study selection is displayed in Figure 1. 

### STUDY CHARACTERISTICS

All included studies^14^ ^-^ ^16^ aimed to investigate the effect of risedronate on orthodontic tooth movement and relapse movement in animals (rats and guinea pigs). Adachi et al.^14^ divided the study into experiment one, in which the anchorage effect of risedronate was evaluated; and experiment two, in which the retentive effect of risedronate was analyzed. In experiment one, the right and left upper first molars of Wistar rats were submitted to orthodontic forces with a standardized expansion spring while undergoing risedronate administration. The risedronate solution was injected into the sub-periosteum region, adjacent to the left upper first molar. The right first molar served as a control, with the injection of 0.9% NaCl solution. In experiment two, the upper right and left first molars were submitted to orthodontic forces for three weeks. The spring was removed, and the administration of risedronate was started (relapse movement). Wu et al.^15^ used 45 female rats (Sprague-Dawley) distributed into three groups: sham (treated with saline), bilaterally ovariectomized rats (OVX, treated with saline), and OVX + intraperitoneally risedronate rats. One month after surgery, a mesial force was applied on the left upper first molar using a nickel-titanium (NiTi) coil. Utari et al.^16^ used an orthodontic appliance to induce a distal orthodontic tooth movement of the lower incisors of guinea pigs (n = 75) up to ±3 mm in length. Risedronate was added to gelatin hydrogel to obtain a semisolid controlled release, and the Bis-CR250 (250 mmol/L) and Bis-CR500 (500 mmol/L) groups (n = 25 per group) were compared to a control group (Bis-CR000, n = 25). Subsequently, risedronate was applied in an intrasulcular manner into the mesial subperiosteal area every three days. After 14 days of stabilization, the apparatus was removed, and the relapse distance between incisors and the osteoclast number with TRAP staining at 0, 3, 7, 14, and 21 days were measured (Table 1).

**Table 1:** General characteristics of the included studies.

Authors (year, country)	Study design	Study subject	Mean age	Study group (number of animals)	Study duration	Primary methods of evaluations	Summary of outcomes
Adachi et al.^14^ (1994, Japan)	Experimental (split-mouth design). Standardized expansion spring, made of 0.012-in Nickel-Titanium (NiTi) wire	126 male Wistar rats	9-10 weeks	Group A - Anchorage effect of risedronate: Group A1: Orthodontic tooth movement effect of risedronate: 50µL of risedronate solution (0.9% NaCl, pH 7.4) at a concentration of either 0 (0.9% NaCl), 125, 250, or 500 µmol/L (left side). Injection of 50µL of 0.9% NaCl solution into the corresponding area used as control (right side). n=41 animals. Group A2: Histological analysis: risedronate at a concentration of 500 µmol/L (left side) and control (0.9% NaCl) (right side) (n=23 animals). Group B - Retentive effect of risedronate: Group B1: Orthodontic tooth movement of risedronate: 50µL of risedronate solution (0.9% NaCl, pH 7.4) at a concentration of either 0 (0.9% NaCl), 125, 250, or 500 µmol/L (left side). Injection of 50 µL of 0.9% NaCl solution into the corresponding area used as control (right side) (n=40). Group B2: Histological analysis: risedronate at a concentration of 500 gmol/L (left side) and control (0.9% NaCl)(right side) (n=22 animals).	Group A: 3 weeks. The administrations started 3 days prior to the set of appliance + application every 3 days. Group A1: 0, 3, 7, 14 or 21 days after application of orthodontic force. Group B: 3 weeks without test drug. Then, the application of risedronate initiated. Group B1: The right and left upper first molars were first moved to the buccal side with the spring without any test drug. Three weeks later, the spring was removed, and administration of risedronate was initiated 0, 3, 7, 14 or 21 days after appliance was removed.	Stone model with sliding calipers: measurement of the distance between the crests of the mesiopalatal cusps of the first molars before and after tooth movement. a = tooth movement or relapse movement on the control side. b = tooth movement or relapse movement on the risedronate-injected side. % inhibition = b/a x 100. Histology: osteoclasts count and measurement of the sizes of active bone-resorptive lacunae.	Group A1: decreased tooth movement on the risedronate-injected side. The percent inhibition (b/a x 100) was 102.1%, 91.2%, 71.1%, and 49.6%X, of the control side at concentrations of 0, 125, 250, and 500µmol/L, respectively. Group A2: Risedronate-treated side had decreased osteoclast count per area at the periods of 3, 7 and 14 days. Decreased percentage of the size of the lacunae and resorptive area on the risedronate-injected side. Group B1: The relapse of the tooth on the risedronate-injected side was significantly less than control. The percent inhibition (b/a x 100) was 96.1%, 79.8%, 73.7%, and 56.7% of the control side at concentrations of 0, 125, 250, and 500 µmol/L, respectively. Group B2: Except on day 14, risedronate had decreased number of osteoclasts in all time points. Sizes of active bone-resorptive lacunae could not be determined.
Wu et al.^15^ (2019, China)	Experimental (split mouth design). Nickel-Titanium alloy closed-coil springs	45 Sprague-Dawley rats	10 weeks	Group A: ovariectomy (saline i.p.). Group B: ovariectomy + risedronate (10ug/kg risedronate dissolved in saline i.p.). Group C: sham (saline i.p.) (n=15 per group)	The administrations started 2 weeks after ovariectomy, and drug administration was every 3 days. On days 3, 7 and 14, five rats from each group were euthanized.	Digital caliper: distance of tooth movement. TRAP staining: osteoclasts count. Immunohistochemical analysis: analysis of RANKL, OPG and CTPSK.	Ovariectomy increased OTM, osteoclasts count, RANKL and CTPSK levels, and decreased OPG levels. Risedronate treatment into the OVX rats reversed all phenotypes.
Utari et al.^16^ (2021, Indonesia)	Experimental (control group and release of risedronate). Nickel-Titanium open coil spring	75 male guinea pigs	5 weeks	Group Bis-CR250 (250mmol/L; 1mg pure risedronate after hydrogel preparation) (n=25) Group Bis-CR500 (500mmol/L; 1.92mg pure risedronate after hydrogel preparation) (n=25) Group Bis-CR000 (control) (n=25)	After reaching ± 3mm of distance between the lower incisor bonding cleat, the distance was maintained for 14 days as a stabilization period, then it was removed. The administrations were every 3 days. On days 3, 7, 14 and 21 relapse movement and interincisal distance were measured.	Sliding caliper: relapse movement and interincisal distance. TRAP staining: osteoclasts count Histological analysis: to calculate the average number of osteoclasts on the mesial (compression side) of the alveolar bone.	Effect to relapse distance: the control had the highest relapse rate at days 14 and 21. There was a significantly less relapse movement in the treatment group on days 14 and 21 compared to control. Bis-CR500 inhibited the relapse movement more effectively than Bis-CR250 on day 21, indicating a dose dependency in the biphosphonate hydrogel application. Number of osteoclasts: osteoclast were abundant along the alveolar bone in Bis-CR000 but decreased in the Bis-CR250 and Bis-CR500 groups.

NaCl = sodium chloride; H.E. = hematoxilin and eosin; TRAP = tartrate-resistant acidic phosphatase; i.p. = intra-peritoneal. RANKL = receptor activator of nuclear factor-kappa β ligand; OPG = osteoprotegerin; CTPSK = cathepsin K; OVX = ovariectomized; OTM = orthodontic tooth movement; % inhibition = b/a x 100: a = tooth movement or relapse movement on the control side. b = tooth movement or relapse movement on the risedronate-injected side.

### STUDY CHARACTERISTICS RELEVANT TO THE APPLICATION OF ORTHODONTIC FORCE

In the study conducted by Adachi et al.^14^ (1994), a 0.012-in NiTi standard and uniform expansion spring was designed and placed in the mouth of each animal between the right and left first molars. An initial force of 165 mN (Milli Newton) was applied on each side and was maintained in the oral cavity by its expansive force. Wu et al.^15^ also used a closed coil made of helical NiTi alloy, and the groups received 30 grams of force (measured by a dynamometer), adjusted by the length of the stainless steel bandage wires (Table 2). Utari et al. ^16^ employed a NiTi open coil spring inserted between the lower incisors using a 0.1 mm stainless steel wire to produce a 0.25 to 1.30 N magnitude of force to the teeth. The spring coil was replaced with a new coil after the incisors reached a ±3-mm inter-incisor distance, which was maintained for 14 days as a stabilization period, at which time risedronate was applied locally (Table 2). 

**Table 2:** Histological and molecular characteristics of orthodontic tooth displacement in the studies included.

Authors (year, country)	Duration	Force	Measurement of tooth displacement	Size of active bone-resorptive lacunae	Number of osteoclasts (TRAP)	Expression levels of RANK ligand, osteoprotegerin and cathepsin K	Statical analysis
Adachi et al.^14^ (1994, Japan)	Experiment 1: 3 weeks of OTM. Experiment 2: 3 weeks of OTM and 3 weeks of relapse	165 mN	The distance between the crests of the mesiopalatal cusps of the first molars before and after tooth movement was measured with sliding calipers using a stone model.	Measured by image analysis.	Active bone-resorptive lacunae were defined as resorptive cavities in which osteoclasts could be seen. The size of a lacuna was expressed as µm^2^/cell.	N.A.	One or two-way analyses of variance. Scheffe F test or the paired t test was used to identify differences between groups. P < 0.05
Wu et al.^15^ (2019, China)	2 weeks	30g	The distance between the mesial wall of the maxillary left second molar and the distal wall of the maxillary left first molar was measured using a digital caliper on days 3, 7 and 14.	It was scanned three visual fields from the alveolar bone tissue on the pressure side of the first molar to calculate the number of osteoclasts after magnification at 400×.	It was selected three visual fields from the alveolar bone tissue on the pressure side of the first molar to calculate the number of osteoclasts after magnification at 400×.	* The positive expression levels of RANK ligand and cathepsin K in the ovariectomy group were stronger than those in the sham group, and the expression of osteoprotegerin in the ovariectomy group was weaker than that in the sham group. * After the ovariectomized rats were injected with risedronate, the positive expression levels of RANK ligand and cathepsin K were decreased, and the expression of osteoprotegerin was increased. * The positive expression levels of RANK ligand and cathepsin K in the ovariectomy+risedronate group were stronger, while the positive expression of osteoprotegerin was weaker than those in the sham group	One-way analysis of variance followed by the Bonferroni test P < 0.05
Utari et al.^16^ (2021, Indonesia)	35 days (14 days of OTM and 21 days of relapse)	0.25 to 1.30 N	Relapse movement and interincisal distance were measured on days 3, 7, 14 and 21 using a sliding caliper	N.A	Data were obtained from five randomized regions of interest from the apical point of the junction, which were taken using a light microscope.	N.A	Shapiro-Wilk test and test of homogeneity of variances. One-way analysis of variance and Kruskal-Wallis test were used to analyze the differences between groups. Least significant difference multiple comparison tests were also applied.

OTM = Orthodontic tooth movement; N = Newtons; N.A. = Not applicable; TRAP = Tartar-resistant acid phosphatase; µm = micrometer.

## STUDY CHARACTERISTICS RELEVANT TO RISEDRONATE ADMINISTRATION

The administration volume of risedronate used by Adachi et al.^14^ was 50 μL of a solution at concentrations of 0 (0.9% NaCl), 125, 250, or 500 µmol/L. The results suggested that the topical application of risedronate may be useful in anchoring and retaining teeth in orthodontic treatment. In the study of Wu et al.^15^, risedronate was diluted in saline and administered intraperitoneally at 10 μg/kg. The authors concluded that risedronate inhibits orthodontic tooth movement in ovariectomized rats and serves to regulate the receptor activator of the nuclear factor-kappa β (RANK)/RANK ligand/osteoprotegerin pathway (Table 2). Similar to Adachi et al.^14^, a topical application of risedronate dissolved in gelatin hydrogel was used by Utari et al.^16^ in the concentrations of 0 (control), 250 mmol/L and 500 mmol/L. Less relapse movement and a decreased osteoclast count was verified by both risedronate treatments, revealing that a dose of 500 mmol/L was more effective (Table 2).

### MAIN STUDY OUTCOME VARIABLES

According to Adachi et al.^14^, the topical administration of risedronate inhibited relapse movement in a dose-dependent manner. Wu et al.^15^ showed decreased orthodontic tooth movement and a reduced number of osteoclasts in the OVX + risedronate group, when compared to the OVX group (Table 3). Utari et al.^16^ verified that topically administered risedronate gelatin hydrogel is highly effective in decreasing the tooth relapse movement and the number of osteoclasts (Table 3).

**Table 3:** Impact of risedronate on OTM (study outcomes).

Authors (year, country)	Results I (Histologic and biologic findings)	Results II (findings related to OTM)	Conclusions
Adachi et al.^14^ (1994, Japan)	* The number of osteoclasts on the risedronate-treated side was significantly less than that on the control side on days 3, 7 and 14 (Control: 0.2, 6.4 and 5.5, and risedronate: 0.3, 3.9 and 5.1, respectively). * The sizes of lacunae on the risedronate-injected side were significantly smaller than those on the control side after day 7. The average sizes of bone-resorptive lacunae on the risedronate-injected side were 37.3%, 38.6%, and 29.2% smaller than those on the control side on days 7, 14, and 21, respectively. * The active bone-resorptive area of the risedronate group was 47.5%, 53.3% and 53.7% decreased compared to control at days 3, 7 and 14, respectively. * In the retentive effect of risedronate except on day 14, risedronate had decreased number of osteoclasts in all time points (control: 4.0, 3.5 and 4.8; and risedronate-injected side 2.8, 1.8 and 2.7 osteoclasts per area on days 3, 7 and 21, respectively)	* Within 3 days, both the control and experimental teeth had moved rapidly about 0.2 mm. * At day 21: the control teeth moved an average of 0.46 mm, whereas experimental teeth moved only 0.23 mm. * After day 10 of force application, the tooth movement on the residronate-injected side was significantly less than that on the control side. The inhibitory effect was dose-depedent. * The relapse of the tooth on the risedronate injected side was significantly less than that on the control side. The inhibitory effect of risedronate was again dose-dependent. At 3 days, there was no statistically difference between control and experimental teeth although had rapidly relapsed about 0.2 mm and 0.23 mm, respectively. At day 14, there was significant difference; and at day 21, the control teeth had relapsed an average of 0.44 mm, whereas experimental teeth had relapsed only 0.24 mm.	No histological differences on the tension were observed between the control side and risedronate-injected side during the experimental period. However, quantitative examinations of bone formation, such as a study of bone formation rates, will be required to determine the precise effect of risedronate on alveolar bone formation incident to tooth movement.
Wu et al.^15^ (2019, China)	* The number of TRAP-positive cells in the ovariectomy group was higher than that in the sham group (P < 0.01 on days 3, 7 and 14). * The number of TRAP-positive cells was reduced after the injection of risedronate into the ovariectomized rats (P < 0.05 on days 3, 7 and 14). * The number of TRAP-positive cells in the ovariectomy + risedronate group was higher than that in the sham group (P < 0.05 on days 3, 7 and 14).	* The orthodontic tooth movement in the ovariectomy group was faster than in the sham group (P < 0.01 on days 3, 7 and 14). * Injection of risedronate into the ovariectomized rats decreased the orthodontic tooth movement velocity (P < 0.01 on days 3 and 14, P < 0.05 on day 7).	Risedronate can inhibit orthodontic tooth movement in ovariectomized rats and may function by regulating the RANK/RANK ligand/osteoprotegerin pathway.
Utari et al.^16^ (2021, Indonesia)	* The release of risedronate with gelatin hydrogel was slower than without the hydrogel carrier; 19.9% to 15.7% in pure 250mg compared to Bis-CR250; and 39.7% to 22.3% in pure 500mg compared to Bis-CR500. * Both Bis-CR250 and Bis-CR500 had no detectable release before 1 hour of immersion. * Osteoclasts were abundant along alveolar bone in Bis-CR000, but decreased in the Bis-CR250 and Bis-CR500 groups, showing the inhibition of osteoclasts activity.	* The control had the highest relapse rate at days 14 and 21. * There was a significantly less relapse movement in the treatment group at days 14 and 21, compared to control. * Bis-CR500 inhibited the relapse movement more effectively than Bis-CR250 on day 21, indicating a dose dependency in the bisphosphonate hydrogel application.	Topically administered bisphosphonate risedronate with gelatin hydrogel effectively decreases the relapse 7 days after the tooth stabilization period in a dose-dependent manner. The developed gelatin hydrogel system is able to deliver the risedronate to a targeted area in a controlled manner and provide local effects, which is useful in orthodontic practice

### RISK OF BIAS ASSESSMENT

The three articles included in the present study^14^ ^-^ ^16^ presented a low risk of bias for sequence generation, baseline characteristics, and incomplete outcome data. For allocation concealment, selective outcome reporting, and other sources of bias, the risk of bias was unclear in all three articles.^14^ ^-^ ^16^ The risk of bias was unclear for random housing and blinding of trial caregivers/researchers in findings from both Adachi et al.^14^ and Utari et al.^16^ studies, and low in findings from of Wu et al.^15^ study. By contrast, the risk of bias was low for random outcome assessment and the blinding of outcome assessors in findings from both Adachi et al.^14^ and Wu et al.^15^ studies, and unclear in findings from Utari et al.^16^ study (Table 4).

**Table 4:** Assessment of risk of bias in the included studies.

	Selection bias			Performance bias		Detection bias		Attrition bias	Reporting bias	Other
	Sequence generation	Baseline characteristics	Allocation concealment	Random housing	Blinding of trial caregivers/researchers	Random outcome assessment	Blinding of outcome assessors	Incomplete outcome data	Selective outcome reporting	Other sources of bias
Adachi et al.^14^ (1994, Japan)	low risk of bias	low risk of bias	unclear risk of bias	unclear risk of bias	unclear risk of bias	low risk of bias	low risk of bias	low risk of bias	unclear risk of bias	unclear risk of bias
Wu et al.^15^ (2019, China)	low risk of bias	low risk of bias	unclear risk of bias	low risk of bias	low risk of bias	low risk of bias	low risk of bias	low risk of bias	unclear risk of bias	unclear risk of bias
Utari et al.^16^ (2021, Indonesia)	low risk of bias	low risk of bias	unclear risk of bias	unclear risk of bias	unclear risk of bias	unclear risk of bias	unclear risk of bias	low risk of bias	unclear risk of bias	unclear risk of bias

High risk of bias = The study does not meet the requirement. Low risk of bias = The study meets the requirement. Unclear risk of bias = It is unclear whether the study meets the requirement.

### CASE REPORT

**Figure 2:**
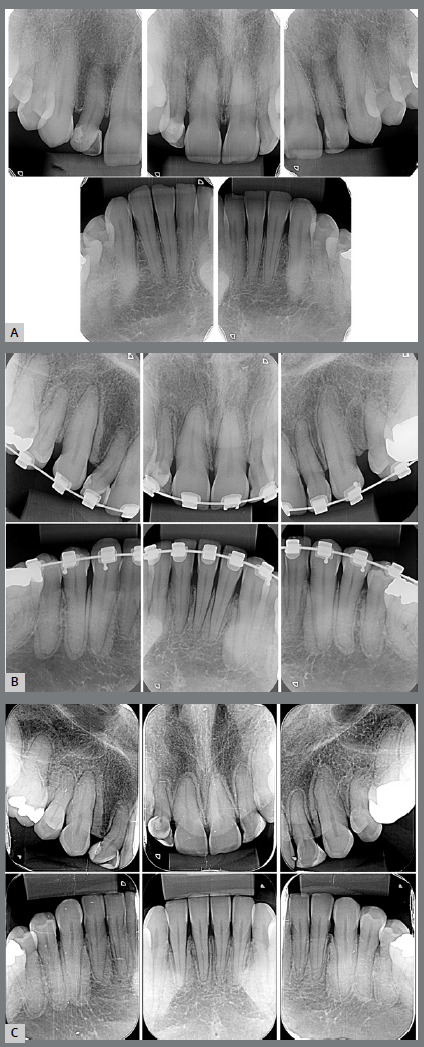
Beginning (A), two years after (B), and final periapical radiographs (C).

**Supplemental Figure 1:**
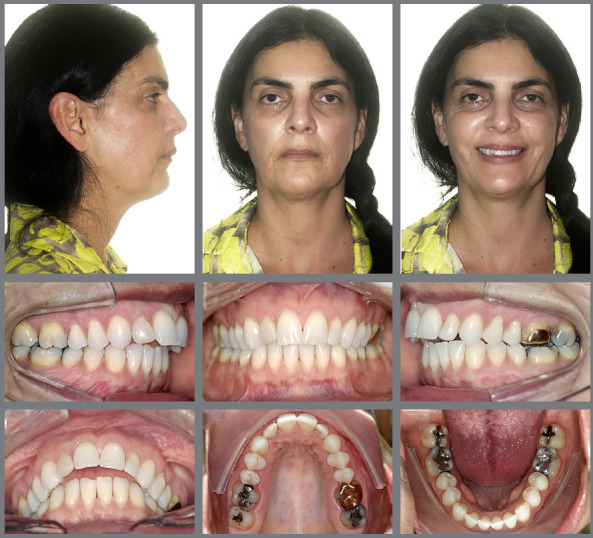
Initial intraoral and extraoral photographs.

**Supplemental Figure 2:**
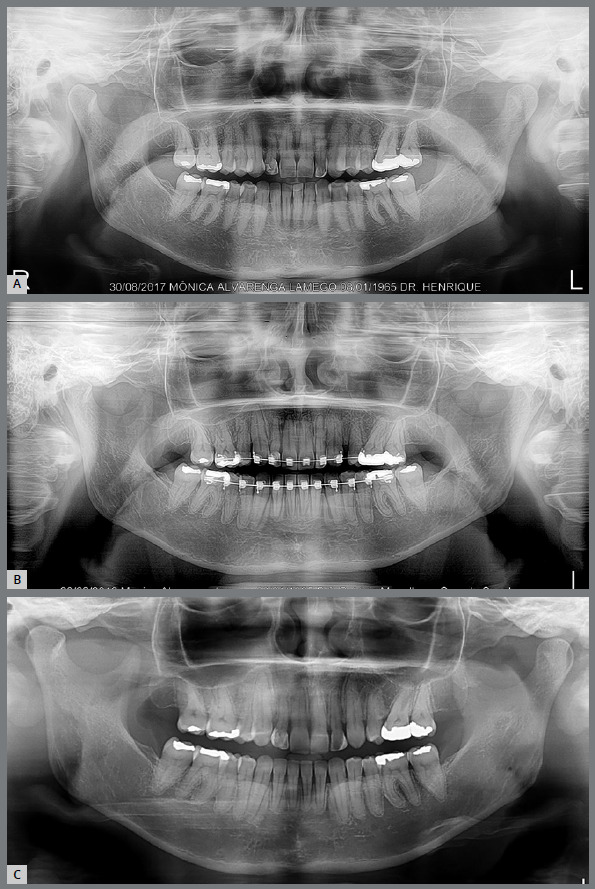
Panoramic radiographs: A) beginning, B) after two years, and C) after orthodontic treatment ( final ).

**Supplemental Figure 3:**
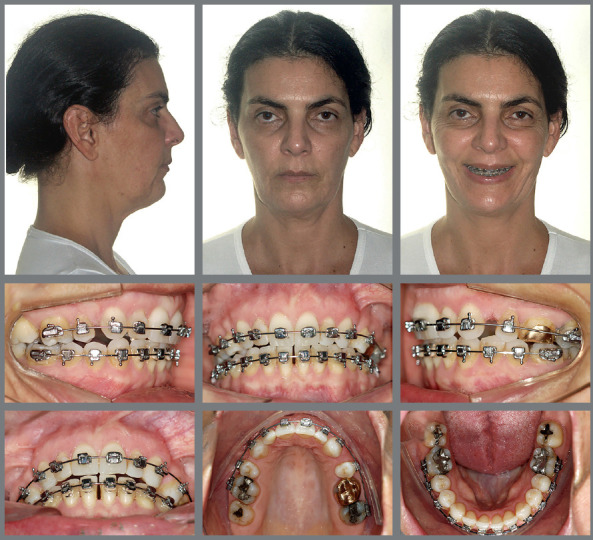
Two years after orthodontic treatment.

A 55-year-old female patient with a diagnosis of osteoporosis was referred to the Orthodontic Clinic at the Federal University of Minas Gerais (Brazil) to undergo treatment with fixed appliances. Her main complaint was the protrusion of her upper anterior teeth and overjet (Suppl. Figs 1 and 2). During anamnesis, the patient reported taking Actonel 150 mg (risedronate) once a month and a calcium supplement daily. She also reported taking Angeliq (drospirenone 2 mg and estradiol 1 mg) to treat the symptoms of menopause, and simvastatin 20 mg to control cholesterol levels, both once a day. All medications had been taken for six years prior to this study. The treatment plan consisted of fixed appliance bonding, alignment, and leveling; the extraction of two upper first premolars; and *en-masse* retraction of the upper anterior teeth for space closure. Two years after the beginning of orthodontic treatment, the *en-masse* retraction was progressing at a slow rate. The orthodontist, who was aware of the intake of bisphosphonate by the patient to treat osteoporosis, requested new orthodontic exams (intraoral and extraoral photographs and radiographic exams), in order to have an overview of treatment progress (Suppl. Fig. 3) and to monitor root resorption (Fig. 2B). The panoramic radiograph revealed an increased mesial inclination and displacement of the upper first molar (Fig 2B). In comparison with pretreatment records, the lower and upper incisors showed a low degree of root resorption (Fig 2B). 

**Supplemental Figure 4:**
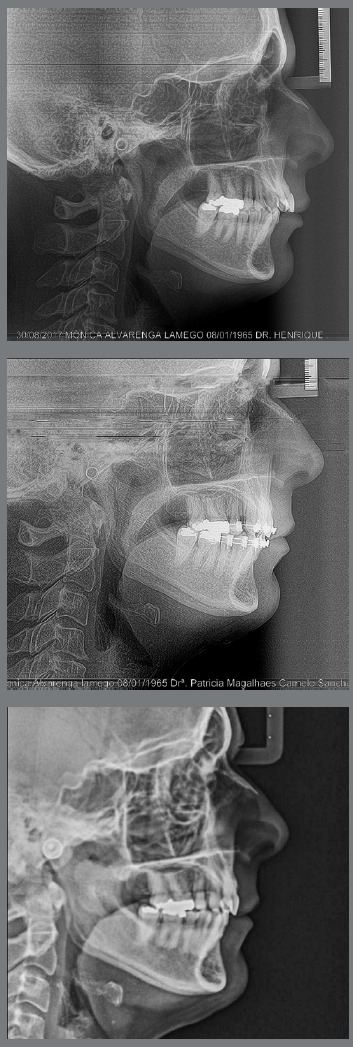
Lateral cephalometric radiographs: initial, after two years, and after 30 months of orthodontic treatment (final ).

**Supplemental Figure 5:**
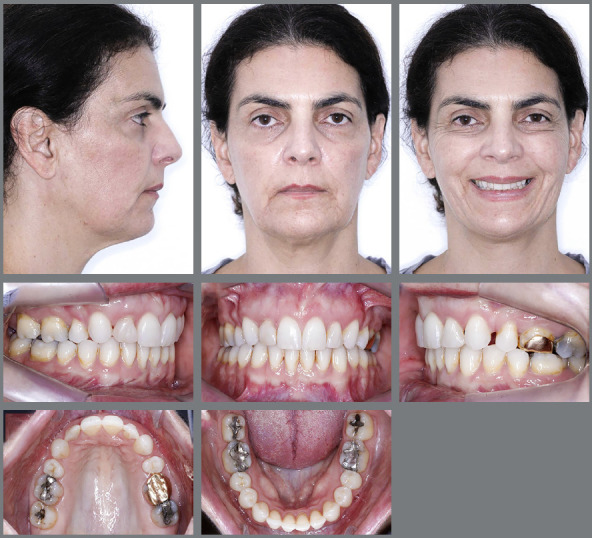
Final intraoral and extraoral photographs.

**Supplemental Figure 6:**
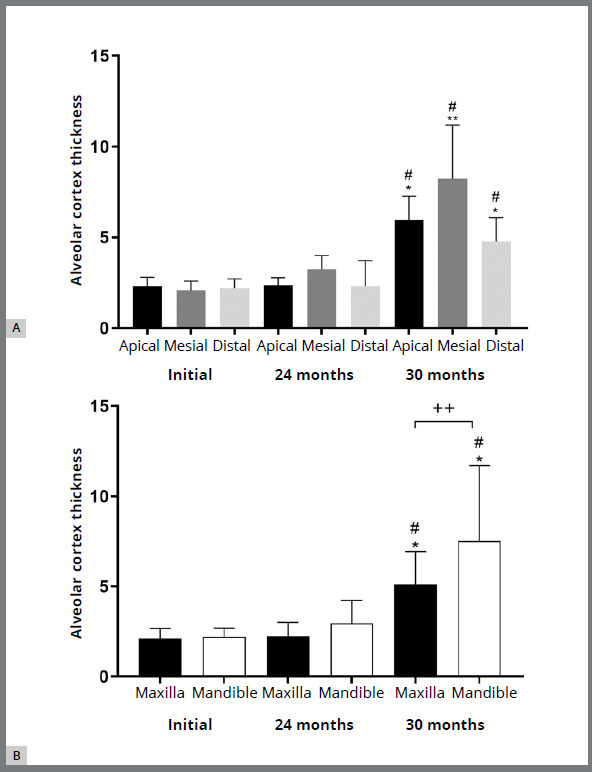
Comparison of the periradicular alveolar bone cortex thickness throughout the three stages of treatment: before the beginning of orthodontic treatment (initial stage), at two years of treatment (24 months), and shortly after fixed appliance debonding within 30 months (final stage). B) Difference between the maxillary and mandibular bones at the different treatment stages. * means different from the beginning. # means different from 24 months. + means different from the maxilla. Analysis of variance (ANOVA), followed by the *post-hoc* Tukey-Kramer test for multiple comparisons. Values of p < 0.05 were considered statistically significant.

The periapical radiographs showed radiopaque areas at the contouring area of all tooth roots, suggesting an increase in bone density in the cortical alveolar bone (Fig 2B). Despite the slow rate of tooth movement, the treatment continued, and nine months after the beginning of the retraction of the upper incisors, the *en-masse* retraction was finished (Suppl. Fig 3). Radiopaque images persisted on the periradicular cortical bone (Fig 2C). At this point, the patient came to her dental appointment with the overjet corrected, half-cusp Class II canine relationship on the left side, and diastema between the maxillary left canine and the maxillary left second pre-molar. The analysis of the lateral cephalometric radiographs showed that the positions of the maxillary incisors were improved at the end of treatment, when compared to the initial treatment phase (Suppl. Fig 4). Radiopaque images persisted on the periradicular cortical bone (Fig 2C, and Suppl. Fig 2C). Although some limitations have occurred over the course of treatment, the patient was satisfied with the achievement of a better occlusion (Suppl. Fig 5). The periradicular alveolar bone cortex density and thickness were analyzed, showing higher density and a larger increase in the thickness of the periradicular alveolar bone cortex in the mandible than in the maxilla over the period of 30 months (Supplemental Material and Suppl. Fig 6).

## DISCUSSION

This systematic review revealed that risedronate impaired orthodontic movement can reduce the number of osteoclasts and the size of resorption gaps in rats. The radiographic analysis of the patient from the aforementioned case report also showed that the chronic intake of risedronate increased the density of both the maxillary and the mandibular periradicular bone, thus impairing orthodontic tooth movement.

Orthodontic tooth movement may be affected by local or systemic medications.^17^ ^-^ ^19^ Bisphosphonates have an inhibitory effect on bone resorption and are successfully administered for the treatment of osseous disorders, such as osteoporosis.^9^ ^,^ ^18^ ^,^ ^20^ Even though the action of bisphosphonate is generally well described in the literature and the effects in human subjects have also been documented,^21^ ^-^ ^23^ studies that specifically evaluate risedronate intake and its impact on orthodontic tooth movement in humans are scarce. Although the results from this study are from animal research, and should thus be viewed with caution, the clinician should nevertheless be aware of the effects of risedronate during alveolar bone remodeling. In this sense, this systematic review in animals associated with the clinical case is of particular relevance.

This is the first systematic review that evaluates the association of the use of risedronate with orthodontic tooth movement, tooth relapse, and the number of osteoclasts in animals. In this systematic review, three articles were selected for inclusion.^14^ ^-^ ^16^ The identified studies were performed exclusively with animals, and no studies were found that assessed orthodontic tooth movement and risedronate in humans. Although the design and sample size of the three included studies were different, the effect of the medication was similar in all studies.

The data obtained in the selected studies demonstrated that the administration of risedronate directly interferes in orthodontic movement^14^ ^,^ ^15^ and in the number of osteoclastic cells,^14^ ^-^ ^16^ which may result in prolonged treatment time and tooth relapse movement,^14^ ^,^ ^16^ with increased retentive effects. In 1994, Adachi et al.^14^ experimentally showed that risedronate reduced the degree of tooth movement and tooth relapse movement in rats in a dose-dependent manner. These effects were also accompanied by a reduction in the number of osteoclasts. 

Corroborating the findings of the present systematic review, *in-vitro* studies have shown that alendronate,^24^ clodronate,^25^ and risedronate^20^ decreased osteoclast differentiation. In addition, studies have already suggested the interference of bisphosphonates (but not risedronate) in human periodontal fibroblasts after mechanical load.^26^ ^,^ ^27^ Risedronate effects reverberate in the osteoblastic proliferation and *in-vitro* differentiation by means of the increase in genes involved in the osteoblastogenesis, such as bone morphogenetic protein-2, bone sialoprotein-II, core-binding factor alpha subunit 1, alkaline phosphatase, osteocalcin, and type 1 collagen.^28^ ^-^ ^30^ The studies included in the systematic review did not analyze the effects of risedronate on the osteoblasts, precluding any comparison between them.

In 1996, Igarashi et al.^31^ investigated the effect of risedronate on root resorption in rats. Their findings showed that the side treated with risedronate showed significantly less root resorption on day 14 and day 21. The authors concluded that risedronate administered topically could prevent root resorption during orthodontic treatment. Though interesting, the outcomes of this study could not be included in our systematic review, since only histological parameters had been evaluated. Results about tooth movement were unavailable.

Findings from Wu et al.^15^ demonstrated that tooth movement and the number of osteoclasts were reduced because of risedronate administration by means of a RANK/RANK ligand/osteoprotegerin pathway in ovariectomized rats. Their findings indicated that risedronate has the potential to prolong orthodontic treatment time and/or establish limitations when compared to treatments during which risedronate had not been administered.^31^

Regarding the effects of the different bisphosphonates on orthodontic movement, the study of Seifi et al.,^32^ in agreement with the results of the present systematic review, demonstrated that zoledronic acid (ZA) is a potent bisphosphonate that suppresses the role of osteoclasts. In their study, ZA did not significantly inhibit orthodontic movement, but rather inhibited root resorption and angiogenesis in rats. Moreover, the continuous use of bisphosphonate suppressed osteoclast activity and preserved the alveolar bone around the roots in a mouse model. After the discontinuation of bisphosphonate, the orthodontic tooth movement remained suppressed.^33^

Bisphosphonates have been used to inhibit bone fragility in patients with osteogenesis imperfecta (OI). Friedrich et al.^34^ concluded that patients with OI using bisphosphonates were able to undergo orthodontic movement with personalized orthodontic forces, applied in a controlled manner, and with longer intervals between orthodontic appointments. Furthermore, an anti-hyperlipidemic drug, such as simvastatin, associated with risedronate, might have further decreased tooth movement effectiveness with increased bone remodeling.^35^ ^,^ ^36^ Sidhu et al.^37^ emphasized the importance of orthodontists being aware of the interactions that drugs used by patients may have in the orthodontic movement process, and indicated drugs and suppressive agents that might reduce bone resorption.

The effects of bisphosphonates on orthodontic tooth movement in osteoporotic patients have already been explored in the literature.^38^ However, the effects of the risedronate should be better elucidated. In accordance with the aforementioned studies, treatment time was enhanced because of the reduced mechanical-load tooth movement in the aforementioned case report. Even though malocclusion was corrected, promoting a better occlusion and solving the patient’s complaint, the continuous risedronate intake reduced the orthodontic tooth movement. These effects were most likely caused as the result of a thickening on the alveolar bone cortex surrounding the roots of the teeth. 

Relapse can occur as a result of forces from the periodontal fibers, which are susceptible to a tooth movement that would return to the pretreatment positions.^39^ The relapse movement is a type of tooth moment that stimulates the osteoclast redistribution in the opposite direction of the orthodontic tooth movement.^40^ Successful orthodontic interventions should be characterized by long-term maintenance, with no relapse. However, orthodontic relapse is an inevitable and unfavorable sequela of orthodontic treatment. The orthodontic relapse, as well as the orthodontic tooth movement rate, can be modulated by different medications,^14^ ^,^ ^16^ ^,^ ^19^ ^,^ ^30^ which can influence the results of the treatment. In addition, Adachi et al.^14^ evaluated both the orthodontic tooth movement (Experiment 1) and the orthodontic relapse (Experiment 2) in rats under risedronate administration. In both experiments, the tooth movement on the risedronate-injected side was significantly less than that on the control side. In this sense, the inclusion of studies showing that risedronate influences not only initial orthodontic tooth movement, but also the orthodontic relapse is of utmost importance.

Based on the analysis of the radiographic periradicular alveolar bone, the bone cortex significantly increased after two years of orthodontic movement, and the effects of risedronate intake were more reactive on the mesial side of the teeth. The orthodontic tooth movement is a result of tension and compression, characterized by bone formation promoted by osteoblasts and resorption promoted by the osteoclasts, respectively.^2^ Risedronate increases osteoblast proliferation and differentiation,^28^ ^-^ ^30^ which can explain the enhanced radiopacity of the mesial side where bone-forming cells prevailed. Therefore, the distal and apical regions were less affected. These findings could be explained by the differential physiological process of the alveolar bone remodeling during orthodontic tooth movement in each specific site.^41^ Moreover, it may be a mechanism of compensation of bone reabsorption on the distal side, since bisphosphonates have an effective inhibitory effect on osteoclasts.^18^ ^,^ ^30^

The more pronounced increase in the periradicular alveolar bone cortex of the mandible in comparison with the maxilla may be associated with the risedronate intake, and may also have followed the natural physiological aspects of those bones, with the mandible having a higher density than the maxilla.^42^ Although there was impairment of tooth movement, a slight amount of root resorption was exhibited after the treatment. These results contrast with those of Zymperdikas et al.,^21^ ^,^ ^43^ who verified a reduction in the prevalence of root resorption after the administration of bisphosphonates. Katz et al.^11^ also revealed a reduced predominance of periapical lesions in osteoporotic patients, particularly among those who took risedronate.

The strengths of the present review include the use of well-established guidelines in an attempt to reduce bias, as well as the exhaustive and comprehensive search strategy (April/2020 up to June/2023). The limitations arise from the number of articles, only three studies in rodents and none in humans; the type of information retrieved; and the means of risedronate administration. The results did not allow us to synthesize the data or perform meta-analyses. Furthermore, it should be emphasized that the information retrieved is not related to humans, a limitation that results in an overall downgrading of the quality of evidence regarding the human context. However, one should not forget that human studies analyzing the effect of risedronate upon orthodontic tooth movement are non-existent. The recommendation for further study is that future investigations, such as a clinical trial, should focus on patients using risedronate under orthodontic treatment.

The clinical decision-making during orthodontic treatment need to be supported by scientific evidence. Orthodontists should perform a careful evaluation of the medical history of the patients undergoing treatment before beginning orthodontic therapy. Given the scarcity of animal studies and the absence of human studies evaluating the effect of risedronate upon orthodontic tooth movement, the randomized clinical trial is regarded as the basis for the evidence-based dentistry paradigm. This insight could be promising for future research in orthodontics.

In summary, the results obtained from the systematic review and the case report indicate that risedronate administration causes impairment of orthodontic tooth movement with compromised clinical outcomes. Although these results are from animal studies and should thus be viewed with caution, the present data may assist oral health practitioners in tailoring specific treatment strategies for each user of this medication while undergoing orthodontic treatment. 

## CONCLUSION

Based on the information compiled in rodents, it can be assumed that the rate of orthodontic tooth movement and tooth relapse movement may be affected by the administration of risedronate.

## Data Availability

Additional data that support the findings of this study are available from the corresponding author, upon reasonable request. Additional informed consent was obtained from all patients for which identifying information is included in this article. All procedures followed were in accordance with the ethical standards of the responsible committee on human experimentation (institutional and national) and with the Helsinki Declaration of 1975, as revised in 2008^5^. Informed consent was obtained from all patients before being included in the study.

## Appendix 1: Search strategies used in the databases.

[Table t1app]

**Table t1app:**

Database	Search strategy
Web of Science	Atelvia OR Risedronate Sodium OR Risedronic Acid Monosodium Salt OR Actonel OR Risedronate OR Bisphosphonate Risedronate Sodium OR Bisphosphonate OR 2-(3-pyridinyl)-1-hydroxyethylidene-bisphosphonate OR 2-(3-pyridinyl)-1-hydroxyethylidenebisphosphonate AND Tooth Movement Technique OR Tooth Movement Techniques OR Orthodontic Tooth Movement OR Orthodontic Tooth Movements OR Tooth Uprighting OR Tooth Uprightings OR Minor Tooth Movement OR Minor Tooth Movements OR Tooth Intrusion OR Tooth Intrusions OR Tooth Depression OR Tooth Depressions OR Orthodontic Treatment OR Orthodontic Therapy OR Orthodontic Movement OR Tooth Movement
Ovid	Atelvia OR Risedronate Sodium OR Risedronic Acid Monosodium Salt OR Actonel OR Risedronate OR Bisphosphonate Risedronate Sodium OR Bisphosphonate OR 2-(3-pyridinyl)-1-hydroxyethylidene-bisphosphonate OR 2-(3-pyridinyl)-1-hydroxyethylidenebisphosphonate AND Tooth Movement Technique OR Tooth Movement Techniques OR Orthodontic Tooth Movement OR Orthodontic Tooth Movements OR Tooth Uprighting OR Tooth Uprightings OR Minor Tooth Movement OR Minor Tooth Movements OR Tooth Intrusion OR Tooth Intrusions OR Tooth Depression OR Tooth Depressions OR Orthodontic Treatment OR Orthodontic Therapy OR Orthodontic Movement OR Tooth Movement
Lilacs	Atelvia OR Risedronate Sodium OR Risedronic Acid Monosodium Salt OR Actonel OR Risedronate OR Bisphosphonate Risedronate Sodium OR Bisphosphonate OR 2-(3-pyridinyl)-1-hydroxyethylidene-bisphosphonate OR 2-(3-pyridinyl)-1-hydroxyethylidenebisphosphonate AND Tooth Movement Technique OR Tooth Movement Techniques OR Orthodontic Tooth Movement OR Orthodontic Tooth Movements OR Tooth Uprighting OR Tooth Uprightings OR Minor Tooth Movement OR Minor Tooth Movements OR Tooth Intrusion OR Tooth Intrusions OR Tooth Depression OR Tooth Depressions OR Orthodontic Treatment OR Orthodontic Therapy OR Orthodontic Movement OR Tooth Movement
Scopus	Atelvia OR “Risedronate Sodium” OR “Risedronic Acid Monosodium Salt” OR Actonel OR Risedronate OR “Bisphosphonate Risedronate Sodium” OR Bisphosphonate OR 2-(3-pyridinyl)-1-hydroxyethylidene-bisphosphonate OR 2-(3-pyridinyl)-1-hydroxyethylidenebisphosphonate AND “Tooth Movement Technique” OR “Tooth Movement Techniques” OR “Orthodontic Tooth Movement” OR “Orthodontic Tooth Movements” OR “Tooth Uprighting” OR “Tooth Uprightings” OR “Minor Tooth Movement” OR “Minor Tooth Movements” OR “Tooth Intrusion” OR “Tooth Intrusions” OR “Tooth Depression” OR “Tooth Depressions” OR “Orthodontic Treatment” OR “Orthodontic Therapy” OR “Orthodontic Movement” OR “Tooth Movement”

## SUPPLEMENTAL MATERIAL

### PERIAPICAL RADIOGRAPHIC ANALYSIS OF THE CASE REPORTED

The bone density was classified, according to Lekholm and Zarb,^1^ as follows: Type I = bone formed of compact and homogeneous tissue with poor blood irrigation; Type II = bone formed of two thick cortical layers, involving a dense trabecular layer; Type III = bone formed by a thin cortical layer, involving a central dense trabecular portion; and Type IV = bone formed by a thin layer of cortical bone, involving trabeculae of low density and reduced resistance.

For the comparison of the periradicular alveolar bone cortex thickness along the three stages of treatment (before orthodontic treatment’s onset [initial stage]; two years of treatment [24 months], and shortly after fixed appliance debonding within 30 months [final stage]). The areas of quantification of the alveolar cortex were measured with Radiocef 6.0 (Radio Memory, Ltda, Belo Horizonte, MG, Brazil). Five measurements were performed on the periapical radiography of each tooth: one at the apex of the tooth using the apical foramen as a reference, two measurements (mesial and distal) at the apical third, 15 mm from the apex, and two measurements at the middle third of each tooth. The teeth involved in the analysis were: upper and lower canine and incisors. While measuring, the radiologist standardized the images at 150 DPI and 0% of magnification. Free brightness, contrast, and zoom tools were also used.

For statistics, the software GraphPad Prism (GraphPad Prism version 8.0c for MAC, GraphPad Software, La Jolla, USA) was used. Data were presented as the mean ± standard deviation (SD). The differences among groups were analyzed by analysis of variance (ANOVA), followed by the *post-hoc* Tukey-Kramer test for multiple comparisons. Values of p < 0.05 were considered statistically significant.

### RESULTS OF THE RADIOGRAPHIC ANALYSIS OF THE CASE REPORTED

On the initial radiographic examination, the patient showed a thin alveolar cortical bone in all teeth and adjacent trabecular bone with Type III density. On the radiographic examination at 24 months, there was a generalized thickening of the alveolar cortex, in particular in the region of premolars and lower canines, being classified as Type II density. A Type I bone density could be seen in all affected regions at the end of the treatment.

No difference between the initial and the 24-month assessment was observed (Suppl. Fig. 6). The final radiographic examination showed a considerable increase of the alveolar cortical bone surrounding the roots of the teeth, in comparison to the initial and the 24-month periods (p<0.0001) (Suppl. Fig. 6), with emphasis on the lower canine region (data not shown). Moreover, the mesial periradicular alveolar bone was increased, compared to the apical and distal areas (p<0.001) (Suppl. Fig. 6). There was a higher increase in the thickness of the periradicular alveolar bone cortex in the mandible than in the maxilla at the period of 30 months (p=0.0001) (Suppl. Fig. 6).

## References

[1] Abreu LG, Santos TR, Melgaço CA, Abreu MHN, Lages EMB, Paiva SM (2018). Impact of orthodontic treatment on adolescents' quality of life a
longitudinal evaluation of treated and untreated individuals. Qual Life Res.

[2] Krishnan V, Davidovitch Z (2006). Cellular, molecular, and tissue-level reactions to orthodontic
force. Am J Orthod Dentofacial Orthop.

[3] Ghoneima AA, Allam ES, Zunt SL, Windsor LJ (2010). Bisphosphonates treatment and orthodontic
considerations. Orthod Craniofac Res.

[4] Jepsen DB, Bergen ES, Pan J, van Poelgeest E, Osman A, Burghle A (2023). Recommendations on deprescribing of bisphosphonates in
osteoporosis guidelines a systematic review. Eur Geriatr Med.

[5] Roelofs AJ, Thompson K, Gordon S, Rogers MJ (2006). Molecular mechanisms of action of bisphosphonates current
status. Clin Cancer Res.

[6] Rogers MJ, Mönkkönen J, Munoz MA (2020). Molecular mechanisms of action of bisphosphonates and new
insights into their effects outside the skeleton. Bone.

[7] Drake MT, Clarke BL, Khosla S (2008). Bisphosphonates mechanism of action and role in clinical
practice. Mayo Clin Proc.

[8] Geusens P, Marin F, Kendler DL, Russo LA, Zerbini CA, Minisola S (2018). Effects of teriparatide compared with risedronate on the risk of
fractures in subgroups of postmenopausal women with severe osteoporosis the
VERO trial. J Bone Miner Res.

[9] Wells GA, Hsieh SC, Zheng C, Peterson J, Tugwell P, Liu W (2022). Risedronate for the primary and secondary prevention of
osteoporotic fractures in postmenopausal women. Cochrane Database Syst Rev.

[10] Figueiredo MA, Medeiros FB, Ortega KL (2020). Osteonecrosis of the jaw in a patient under treatment of
osteoporosis with oral bisphosphonate. Autops Case Rep.

[11] Katz J, Rotstein I (2021). Prevalence of periapical lesions in patients with
osteoporosis. J Endod.

[12] Fujita Y, Watanabe K, Uchikanbori S, Maki K (2011). Effects of risedronate on cortical and trabecular bone of the
mandible in glucocorticoid-treated growing rats. Am J Orthod Dentofacial Orthop.

[13] Hooijmans CR, Rovers MM, Vries RB, Leenaars M, Ritskes-Hoitinga M, Langendam MW (2014). SYRCLE's risk of bias tool for animal studies. BMC Med Res Methodol.

[14] Adachi H, Igarashi K, Mitani H, Shinoda H (1994). Effects of topical administration of a bisphosphonate
(risedronate) on orthodontic tooth movements in rats. J Dent Res.

[15] Wu D, Meng B, Cheng Y, Gan L, Huang P, Cao Y (2019). The effect of risedronate on orthodontic tooth movement in
ovariectomized rats. Arch Oral Biol.

[16] Utari TR, Ana ID, Pudyani PS, Asmara W (2021). The intrasulcular application effect of bisphosphonate hydrogel
toward osteoclast activity and relapse movement. Saudi Dent J.

[17] Makrygiannakis MA, Kaklamanos EG, Athanasiou AE (2019). Effects of systemic medication on root resorption associated with
orthodontic tooth movement a systematic review of animal
studies. Eur J Orthod.

[18] Kouskoura T, Katsaros C, von Gunten S (2017). The potential use of pharmacological agents to modulate
Orthodontic Tooth Movement (OTM). Front Physiol.

[19] Kaklamanos EG, Makrygiannakis MA, Athanasiou AE (2021). Could medications and biologic factors affect post-orthodontic
tooth movement changes A systematic review of animal studies. Orthod Craniofac Res.

[20] Meng B, Yang B, Qu Y, Liu Y, Wu D, Fu C (2023). Dual role of interleukin-20 in different stages of osteoclast
differentiation and its osteoimmune regulation during alveolar bone
remodeling. Int J Mol Sci.

[21] Zymperdikas VF, Yavropoulou MP, Kaklamanos EG, Papadopoulos MA (2020). Effects of systematic bisphosphonate use in patients under
orthodontic treatment a systematic review. Eur J Orthod.

[22] Makrygiannakis MA, Kaklamanos EG, Athanasiou AE (2019). Medication and orthodontic tooth movement. J Orthod.

[23] Kaklamanos EG, Makrygiannakis MA, Athanasiou AE (2020). Does medication administration affect the rate of orthodontic
tooth movement and root resorption development in humans A systematic
review. Eur J Orthod.

[24] Kim SE, Suh DH, Yun YP, Lee JY, Park K, Chung JY (2012). Local delivery of alendronate eluting chitosan scaffold can
effectively increase osteoblast functions and inhibit osteoclast
differentiation. J Mater Sci Mater Med.

[25] Liu L, Igarashi K, Kanzaki H, Chiba M, Shinoda H, Mitani H (2006). Clodronate inhibits PGE(2) production in compressed periodontal
ligament cells. J Dent Res.

[26] Grimm S, Wolff E, Walter C, Pabst AM, Mundethu A, Jacobs C (2020). Influence of clodronate and compressive force on IL-1ß-stimulated
human periodontal ligament fibroblasts. Clin Oral Investig.

[27] Jacobs C, Walter C, Ziebart T, Dirks I, Schramm S, Grimm S (2015). Mechanical loading influences the effects of bisphosphonates on
human periodontal ligament fibroblasts. Clin Oral Investig.

[28] von Knoch F, Jaquiery C, Kowalsky M, Schaeren S, Alabre C, Martin I (2005). Effects of bisphosphonates on proliferation and osteoblast
differentiation of human bone marrow stromal cells. Biomaterials.

[29] Im GI, Qureshi SA, Kenney J, Rubash HE, Shanbhag AS (2004). Osteoblast proliferation and maturation by
bisphosphonates. Biomaterials.

[30] Utari TR, Pudyani PS, Ana ID, Asmara W (2022). The potential of bisphosphonate risedronate hydrogel in
preventing relapse movement. Cumhuriyet Dent J.

[31] Igarashi K, Adachi H, Mitani H, Shinoda H (1996). Inhibitory effect of the topical administration of a
bisphosphonate (risedronate) on root resorption incident to orthodontic
tooth movement in rats. J Dent Res.

[32] Seifi M, Asefi S, Hatamifard G, Lotfi A (2017). Effect of local injection of Zolena, zoledronic acid made in
Iran, on orthodontic tooth movement and root and bone resorption in
rats. J Dent Res Dent Clin Dent Prospects.

[33] Minamoto C, Miyazawa K, Tabuchi M, Hirano M, Mizuno M, Yoshizako M (2020). Alteration of tooth movement by reveromycin A in
osteoprotegerin-deficient mice. Am J Orthod Dentofacial Orthop.

[34] Friedrich RE, Scheuer HA, Höltje W (2019). The effect of bisphosphonate medication on orthodontics and
orthognathic surgery in patients with osteogenesis
imperfecta. GMS Interdiscip Plast Reconstr Surg DGPW.

[35] AlSwafeeri H, ElKenany W, Mowafy M, Karam S (2019). Effect of local administration of simvastatin on orthodontic
tooth movement in rabbits. Am J Orthod Dentofacial Orthop.

[36] Kommuri K, Javed F, Akram Z, Khan J (2020). Effect of statins on orthodontic tooth movement a systematic
review of animal and clinical studies. Arch Oral Biol.

[37] Sidhu S (2019). Drug induced orthodontic tooth movement. J Advanc Med Dent Sci Res.

[38] Arbelaez ML, Garcia SMV, Lopez JP, Avila D, Munevar JC, Pauwels A (2018). Effect of bisphosphonates on orthodontic tooth movement in
osteoporotic patients a review. J World Fed Orthod.

[39] Johnston CD, Littlewood SJ (2015). Retention in orthodontics. Br Dent J.

[40] Thilander B (2000). Orthodontic relapse versus natural development. Am J Orthod Dentofacial Orthop.

[41] Guo R, Zhang L, Hu M, Huang Y, Li W (2021). Alveolar bone changes in maxillary and mandibular anterior teeth
during orthodontic treatment a systematic review and
meta-analysis. Orthod Craniofac Res.

[42] Truhlar RS, Orenstein IH, Morris HF, Ochi S (1997). Distribution of bone quality in patients receiving endosseous
dental implants. J Oral Maxillofac Surg.

[43] Zymperdikas VF, Yavropoulou MP, Kaklamanos EG, Papadopoulos MA (2021). Bisphosphonates as supplement to dental treatment a network
meta-analysis. J Dent Res.

[44] Page MJ, McKenzie JE, Bossuyt PM, Boutron I, Hoffmann TC, Mulrow CD (2021). The PRISMA 2020 statement an updated guideline for reporting
systematic reviews. Int J Surg.

